# Microwave treatment of rice bran and its effect on phytochemical content and antioxidant activity

**DOI:** 10.1038/s41598-022-11744-1

**Published:** 2022-05-11

**Authors:** Piramon Pokkanta, Jitkunya Yuenyong, Sugunya Mahatheeranont, Sudarat Jiamyangyuen, Phumon Sookwong

**Affiliations:** 1 Rice and Cereal Chemistry Research Laboratory, Department of Chemistry, Faculty of Science, Chiang Mai University, Chiang Mai, 50200 Thailand; 2 PhD’s Degree Program in Chemistry, Faculty of Science, Chiang Mai University, Chiang Mai, 50200 Thailand; 3 Research Center On Chemistry for Development of Health Promoting Products From Northern Resources, Chiang Mai University, Chiang Mai, 50200 Thailand; 4 Center of Excellence for Innovation in Chemistry, Faculty of Science, Chiang Mai University, Chiang Mai, 50200 Thailand; 5 Rice and Bioactive Compound Analysis, Department of Agro-Industry, Faculty of Agriculture, Natural Resources and Environment, Naresuan University, Phitsanulok, 65000 Thailand

**Keywords:** Plant sciences, Chemistry, Biochemistry, Nutrition

## Abstract

An alternative approach for rice bran stabilization is microwave treatment. However, the effects of the microwave treatment on the contents of bioactive compounds and antioxidant activities of the rice bran have rarely been reported in detail. In this study, microwave pretreatment (130–880 W for 0.5–5.0 min) of rice bran was proposed where the antioxidant activity, total flavonoids, and total phenolic contents were determined using UV–Vis spectrometry. Tocols, γ-oryzanols, squalene, phytosterols and phenolic compounds were quantified using high-performance liquid chromatography. The results showed an increase in the antioxidant activity (0.5 folds), total phenolic contents (1.3 folds), total flavonoid contents (0.9 folds), total tocols (2.6 folds), total γ-oryzanols (1.6 folds), and total phytosterols (1.4 folds). Phytochemicals were enhanced, especially trans-*p*-coumaric acid (10.3 folds) and kaempferol (8.6 folds). The microwave treatment at 440 W for 2.5 min provided the best contents of the bioactive compounds and antioxidant activity. This work revealed the microwave treatment as a potential tool for stabilizing rice bran and increasing the usability of its phytochemicals, which applies to several industries concerning the use of rice bran as an ingredient.

## Introduction

Recently, microwave treatment (MWT) has been introduced as an effective tool to stabilize plant seeds including sunflower, apricot kernels, and poppy seeds^1,2^. The stabilization process is an important procedure to deactivate lipase activity present in raw materials, thereby preventing them from the hydrolysis of triglycerides into glycerol and free fatty acids. Consequently, the latter compounds are prone to oxidation reactions, leading to unwanted characteristics during storage (hydrolytic rancidity, pH reduction, and soapy taste products)^3^. Several advantages of microwave stabilization of rice bran (RB), compared with other methods such as steaming, parboiling, autoclave heating, roasting, enzymatic treatment, or infrared radiations, include faster treatment time, greater convenience, better cost performance, instantaneous control as well as increasing oil yield and antioxidant activity^4–6^.

Bioactive compounds present in RB such as vitamin E (tocols), γ-oryzanols, phenolic compounds, and plant sterols have received increasing attention due to their therapeutic properties. Their effectiveness in the treatment of coronary heart diseases, serum hypercholesterolemia, nerve imbalance, hyperlipidemia, hyperglycemia, type I and type II diabetes mellitus, inflammatory, regulation of blood clotting, and cancer has been reported^7–10^. To effectively utilize the phytochemicals in the RB, stabilization processes after the rice milling has been recommended. Despite their main purpose of deactivation of lipase, stabilization processes also affect physical and chemical properties of RB^11^. For instance, roasting and enzymatic pretreatment on the RB reduced the yield of RB oil extracted as compared to the control^4^. The heat stabilizations with cooking (parboiling, steaming) led to the high loss of nutrients and antioxidants^12,13^. In the past, studies have shown the effects of RB stabilization on chemical changes. However, the effect of microwave stabilization has been marginally investigated, specifically in terms of bioactive compounds.

Therefore, this study aimed to investigate the influence of MWT on the antioxidant activity and contents of several bioactive compounds using spectrophotometry, and high-performance liquid chromatography (HPLC) techniques. The knowledge derived from this work would aid useful for several industries concerning the use of RB as an ingredient in industrialized processes.

## Results and discussion

### Total phenolic content, total flavonoid content and antioxidant activity

The total phenolic content (TPC), total flavonoid content (TFC), and antioxidant activity (AA) of RB roasted in the microwave oven (MWO) are shown in Table 1. The TPC in RBs varied from 11.01 to 39.62 µg of gallic acid equivalents per g of dry weight (μg GAE/g) (Table 1) whilst the control contained 17.14 μg GAE/g. At low MW power (130 W), the oxidase and lipase could not be deactivated properly. However, the MW-induced heat was able to facilitate the oxidation and enzymatic degradation, leading to a reduction of TPCs (11.01 to 17.08 μg GAE/g). MW power of 260 and 440 W were more suitable for RB stabilization, where an increase in TPCs was observed. The highest TPC was 39.62 μg GAE/g in the bran stabilized at 440 W for 2.5 min (1.3 folds). MW power at 620 W (14.15 to 15.89 μg GAE/g) and 880 W (12.67 to 16.72 μg GAE/g) showed a reduction in the TPCs, which might be owing to the highly generated heat in the MWO destroying the phenolic compounds. The TFCs ranged from 3.88 to 11.61 µg of catechol equivalents per g of dry weight (μg CE/g) (Table 1). The control contained 6.14 μg CE/g, and the highest content was 11.61 μg CE/g in the RB treated at 440 W for 2.5 min. TFCs were increased when treated with 130 W (0.5, 1.0, and 1.5 min), 260 W (0.5 and 1.0 min), 440 W (all treatments), and 620 W (1.0 and 1.5 min). Specifically, TFCs were highest when MW power of 440 W was applied (8.24–11.61 µg CE/g), whilst MWT at 880 W caused a reduction in the TFCs compared to the control. A previous study reported a similar trend for TFCs in apricot kernels roasted in MWO^1^. AA of the RB samples was evaluated using free radical DPPH scavenging assay, and expressed as DPPH scavenging percentage (%). AA of the control was 58.87%, and it was increased after the MWT, especially after roasting at 440 W (79.86–88.94%) and 880 W (61.81–77.03%) (Table 1). The highest value was observed in the RB treated at 440 W for 2.5 min (88.94%, increased 0.5 folds). The increase in AA after MWT agreed with a study by Wijesundera (2008), who used MWT on canola and mustard seed^14^. The increase in AA, TPC, and TFC is involved with the capability to release phenolic compounds and other phytochemical compounds from bound structures after breaking of bonds by the MWT^15^. Nevertheless, using long exposure time and high temperature in the MWO could significantly destroy the TPC and TFC in cereals^1,2^. Generally, MWT at 440 W for 2.5 min was the condition that provided the highest values of TFC, TPC, and AA of the RB. The MWT was reported to be effective in stabilizing RB and inhibiting lipase activity^1^, therefore, obtaining desirable phytochemical properties in RB for human consumption.

**Table 1:** Total phenolic, flavonoid, and antioxidant activity of rice bran treated in microwave oven.

Power/time	Control	130 W							260 W					
		0.5 min	1 min	1.5 min	2 min	2.5 min	3 min	5 min	0.5 min	1 min	1.5 min	2 min	2.5 min	3 min
Total phenolic (μg GAE g)	17.14 ± 0.04i	16.04 ± 0.12j	15.58 ± 0.15j	12.25 ± 0.09m	15.35 ± 0.11jk	11.01 ± 0.10n	12.80 ± 0.11m	17.08 ± 0.03i	19.31 ± 0.01g	17.24 ± 0.01i	20.04 ± 0.01g	25.65 ± 0.01e	18.40 ± 0.01h	23.49 ± 0.01f
Flavonoid (μg CE/g)	6.14 ± 0.02jk	6.44 ± 0.03hi	6.33 ± 0.02i	6.65 ± 0.01g	5.75 ± 0.01o	5.56 ± 0.01m	4.98 ± 0.02lm	5.75 ± 0.01l	6.27 ± 0.01ij	6.70 ± 0.01g	5.59 ± 0.01lm	4.90 ± 0.02op	4.76 ± 0.01p	4.38 ± 0.01q
Antioxidant activity (DPPH scavenging %)	58.87 ± 0.17t	56.09 ± 0.12u	34.80 ± 0.25x	45.36 ± 0.26v	34.26 ± 0.23y	19.61 ± 0.11a	25.87 ± 0.18z	36.55 ± 0.05w	70.62 ± 0.25i	67.68 ± 0.23j	66.39 ± 0.02m	61.66 ± 0.25s	62.65 ± 0.26p	65.62 ± 0.28n

Power/time	440 W					620 W				880 W				
	0.5 min	1 min	1.5 min	2 min	2.5 min	0.5 min	1 min	1.5 min	2 min	0.5 min	1 min	1.5 min	2 min	
Total phenolic (μg GAE/g)	19.77 ± 0.05g	32.75 ± 0.00c	32.95 ± 0.10bc	33.62 ± 0.07b	39.62 ± 0.12a	15.60 ± 0.02j	15.89 ± 0.10j	14.56 ± 0.02j	14.15 ± 0.08 l	15.81 ± 0.09e	16.72 ± 0.01d	14.27 ± 0.02 l	12.67 ± 0.07m	
Flavonoid (μg CE/g)	8.24 ± 0.01e	9.34 ± 0.01d	10.48 ± 0.03b	10.02 ± 0.04c	11.61 ± 0.02a	6.04 ± 0.01lk	6.54 ± 0.01gh	7.75 ± 0.02g	4.56 ± 0.03q	4.76 ± 0.01p	5.34 ± 0.01n	4.15 ± 0.01r	3.88 ± 0.01s	
Antioxidant activity (DPPH scavenging %)	88.71 ± 0.23b	79.86 ± 0.08e	82.75 ± 0.14d	87.79 ± 0.27c	88.94 ± 0.27a	67.42 ± 032l	73.67 ± 0.11 g	62.95 ± 0.24q	62.65 ± 0.24o	67.53 ± 0.12k	77.03 ± 0.22h	71.35 ± 0.27f	61.81 ± 0.11r	

Rice bran was burnt partially (treatment in 260 W for 5 min, 440 W for 3 and 5 min as well as 620 W and 880 W for 2.5, 3, 5 min).

Values are means ± standard deviations (*n* = 3).

### Phenolic compounds content

Eleven phenolic compounds in the RB sample were analyzed with HPLC (Table 2), and the chromatographic elution order was gallic acid, protocatechuic acid, 4-hydroxybenzoic acid, catechin, vanillic acid, chlorogenic acid, caffeic acid, kaempferol, epigallocatechin, trans-*p*-coumaric acid, and sinapic acid (Supplementary Fig. 1). It was interesting to note that the content of trans-*p*-coumaric acid had increased by 10.3 folds from 1.82 μg/g in the control to 20.53 μg/g after roasting at 440 W for 1.5 min. Kaempferol, a curative agent against cancer cell growth, was increased markedly by up to 9.6 folds (6.53 μg/g) after MWT at 440 W for 2.0 min. The MWT at 880 W showed a negative effect which reduced the catechin content (18.67–22.86 μg/g) from the control (23.30 μg/g). Furthermore, a reduction in gallic acid content was obtained after MWT at 130 W (3 and 5 min) and 880 W (1.5 and 2 min). The chlorogenic acid, caffeic acid, epigallocatechin, and sinapic acid in RBs showed positive changes when applied with the microwave roasting process. Protocatechuic acid and vanillic acid were the only two phenolic compounds that decreased after MWT. Similar observations were reported in a study of blue poppy seed^16^. The combination of high power and long operation time exhibited a significant decrease in the phenolic content, which might be due to the partial burning of the RB and thermal damage from hydrothermal treatments on nutraceutical contents of RB as reported by Prateep (2014)^13^. Based on our results, MWT at 440 W for 1.5 min was the best MWO setting to increase the overall phenolics content.

**Table 2:** Phenolic compounds of rice bran treated in microwave oven (µg/g).

Phenolic compound	Control	130 W							260 W					
		0.5 min	1 min	1.5 min	2 min	2.5 min	3 min	5 min	0.5 min	1 min	1.5 min	2 min	2.5 min	3 min
Gallic acid	25.21 ± 0.32l	25.82 ± 0.06m	32.67 ± 0.10cd	30.77 ± 0.34h	29.77 ± 0.30i	28.58 ± 0.05h	10.81 ± 0.06o	8.48 ± 0.16b	29.68 ± 0.29i	32.31 ± 0.07ef	32.70 ± 0.08c	34.42 ± 0.11a	34.24 ± 0.36a	30.66 ± 0.25h
Protocatechuic acid	5.97 ± 0.33a	1.64 ± 0.48i	4.01 ± 0.15bc	4.86 ± 0.03b	4.51 ± 0.16bc	3.64 ± 0.09h	2.59 ± 0.31bc	1.78 ± 0.27i	3.00 ± 0.20g	4.40 ± 0.38cd	4.42 ± 0.07e	2.93 ± 0.55gh	2.51 ± 0.06bc	2.13 ± 0.22fg
4-Hydroxybenzoic acid	2.79 ± 0.37fg	1.70 ± 0.60jk	0.31 ± 0.28p	0.96 ± 0.04lm	1.60 ± 0.34jk	2.89 ± 0.23 fg	2.04 ± 0.12ij	1.64 ± 0.33de	4.20 ± 0.18c	2.70 ± 0.14gh	3.24 ± 0.03ef	4.02 ± 0.13cd	3.92 ± 0.26cd	1.70 ± 0.34b
Catechin	23.30 ± 0.29ij	28.86 ± 0.23 l	36.44 ± 0.46d	42.4 ± 0.52f.	39.71 ± 0.45d	35.05 ± 1.21gh	34.26 ± 0.95 m	31.42 ± 0.14c	32.82 ± 0.62o	42.54 ± 0.20a	44.56 ± 0.33a	38.16 ± 0.58o	36.17 ± 0.53ab	35.43 ± 0.57f
Vanillic acid	4.04 ± 0.27a	1.18 ± 0.46bc	2.06 ± 0.36a	3.06 ± 0.36a	3.16 ± 0.92cd	2.35 ± 0.21ef	2.14 ± 0.03k	2.05 ± 0.23bc	1.78 ± 0.08gh	2.24 ± 0.50e	3.26 ± 0.16b	3.09 ± 0.32b	1.81 ± 0.37gh	1.90 ± 0.10fg
Chlorogenic acid	4.67 ± 0.40h	4.75 ± 0.42kl	8.68 ± 0.11f	9.29 ± 0.07e	6.50 ± 0.45h	6.28 ± 0.37h	4.82 ± 0.09p	1.18 ± 0.49h	5.36 ± 0.23i	9.10 ± 0.34ij	11.59 ± 0.02g	7.59 ± 0.12g	6.06 ± 0.46h	1.18 ± 0.17jk
Caffeic acid	0.95 ± 0.28jkl	1.10 ± 0.34b	1.31 ± 0.33ab	2.40 ± 0.21d	2.70 ± 0.26a	2.08 ± 0.14jkl	0.93 ± 0.08 l	0.69 ± 0.10bc	1.81 ± 0.07cd	2.08 ± 0.07b	2.42 ± 0.03a	1.78 ± 0.10d	1.68 ± 0.16de	1.44 ± 0.16fg
Kaempferol	0.68 ± 0.03p	1.12 ± 0.02m	2.08 ± 0.01j	2.69 ± 0.06o	4.62 ± 0.02c	2.53 ± 0.01r	1.54 ± 0.06k	1.18 ± 0.00f	3.67 ± 0.02g	2.56 ± 0.00i	2.73 ± 0.01h	5.98 ± 0.00e	3.19 ± 0.06l	0.18 ± 0.07t
Epigallocatechin	2.56 ± 0.06ij	2.39 ± 0.16m	2.18 ± 0.30q	1.88 ± 0.15x	1.90 ± 0.20w	2.31 ± 0.05o	2.04 ± 0.02u	2.25 ± 0.06p	2.08 ± 0.04g	2.57 ± 0.03t	2.73 ± 0.07i	3.78 ± 0.04c	2.50 ± 0.10k	2.19 ± 0.05q
Trans-*p*-coumaric acid	1.82 ± 0.02q	13.62 ± 0.05k	17.90 ± 0.14c	17.82 ± 0.11h	17.30 ± 0.05i	16.55 ± 0.04j	5.32 ± 0.12o	5.23 ± 0.19b	17.77 ± 0.09h	18.54 ± 0.08f	18.86 ± 0.20e	19.35 ± 0.07d	19.59 ± 0.10c	16.62 ± 0.03j
Sinapic acid	3.44 ± 0.03f	3.97 ± 0.00de	5.05 ± 0.07c	5.20 ± 0.07c	5.76 ± 0.09e	5.90 ± 0.11e	6.43 ± 0.45a	5.03 ± 0.16c	4.16 ± 0.15d	6.19 ± 0.14b	6.46 ± 0.13a	6.97 ± 0.21de	6.49 ± 0.06f	2.32 ± 0.03hi
Total phenolics	75.43 ± 0.22g	86.15 ± 0.26gh	112.69 ± 0.21bc	116.47 ± 0.18c	117.53 ± 0.30c	108.16 ± 0.23e	72.92 ± 0.21lm	60.93 ± 0.19bc	106.33 ± 0.18h	125.23 ± 0.14bc	132.97 ± 0.10a	128.07 ± 0.20 fg	118.16 ± 0.23b	95.71 ± 0.18de

Phenolic compound	440 W					620 W				880 W				
	0.5 min	1 min	1.5 min	2 min	2.5 min	0.5 min	1 min	1.5 min	2 min	0.5 min	1 min	1.5 min	2 min	
Gallic acid	30.65 ± 0.05h	31.26 ± 0.11g	32.90 ± 0.30cd	31.26 ± 0.09g	30.26 ± 0.41ef	29.55 ± 0.17j	31.56 ± 0.13k	23.42 ± 0.13de	13.42 ± 0.29f.	29.34 ± 0.11ij	29.74 ± 0.15g	22.55 ± 0.03p	11.99 ± 0.22n	
Protocatechuic acid	4.40 ± 0.37cd	4.57 ± 0.10m	4.86 ± 0.03kl	2.10 ± 0.15d	2.03 ± 0.19bc	1.17 ± 0.25jk	1.49 ± 0.04ij	1.07 ± 0.15kl	0.78 ± 0.14lm	0.89 ± 0.15klm	0.87 ± 0.29klm	0.56 ± 0.06 m	0.44 ± 0.13ef	
4-Hydroxybenzoic acid	2.23 ± 0.31hi	3.85 ± 0.13cd	6.00 ± 0.53a	1.29 ± 0.47kl	0.65 ± 0.04nop	0.80 ± 0.07mno	1.27 ± 0.13klm	1.00 ± 0.04kl	0.89 ± 0.12lmno	0.28 ± 0.07p	0.44 ± 0.41op	3.71 ± 0.18de	0.48 ± 0.12op	
Catechin	34.59 ± 0.10g	36.73 ± 0.70j	45.68 ± 0.03b	33.70 ± 0.63hi	6.10 ± 0.03p	32.45 ± 0.34l	34.25 ± 0.19k	36.25 ± 0.57e	33.20 ± 0.06q	20.27 ± 0.13o	21.25 ± 0.44n	22.86 ± 0.22p	18.67 ± 0.10q	
Vanillic acid	2.58 ± 0.13de	3.39 ± 0.16k	3.94 ± 0.19hi	1.72 ± 0.47k	1.51 ± 0.00hi	1.92 ± 0.28j	2.21 ± 0.01k	1.15 ± 0.19k	0.60 ± 0.14k	1.09 ± 0.12ij	1.29 ± 0.33ij	0.39 ± 0.27k	0.22 ± 0.07k	
Chlorogenic acid	5.99 ± 0.43ij	10.32 ± 0.03 lm	14.86 ± 0.28jk	4.75 ± 0.24n	2.19 ± 0.07o	7.16 ± 0.11p	18.90 ± 0.33ef	16.13 ± 0.16d	4.13 ± 0.42 m	10.47 ± 0.23c	15.14 ± 0.10b	17.06 ± 0.06a	1.32 ± 0.09p	
Caffeic acid	1.38 ± 0.12hi	2.48 ± 0.05ef	2.80 ± 0.03de	1.72 ± 0.11de	1.37 ± 0.01gh	1.16 ± 0.19kl	1.20 ± 0.03kl	1.83 ± 0.11gh	0.92 ± 0.32jk	0.70 ± 0.24gh	0.88 ± 0.07l	1.07 ± 0.19kl	0.90 ± 0.13ij	
Kaempferol	2.06 ± 0.02u	4.01 ± 0.01d	5.19 ± 0.02b	6.53 ± 0.09a	0.02 ± 0.02w	0.70 ± 0.01o	0.87 ± 0.01n	0.70 ± 0.05x	0.42 ± 0.09x	0.37 ± 0.07s	0.52 ± 0.02r	0.46 ± 0.00q	0.04 ± 0.00v	
Epigallocatechin	3.65 ± 0.01h	3.74 ± 0.15d	3.36 ± 0.14e	2.94 ± 0.06f	2.11 ± 0.04ls	4.11 ± 0.13a	3.91 ± 0.19b	2.15 ± 0.04lr	1.15 ± 0.37y	3.97 ± 0.04v	2.43 ± 0.06l	2.55 ± 0.14jn	1.04 ± 0.18y	
Trans-*p*-coumaric acid	18.31 ± 0.24g	19.76 ± 0.13h	20.53 ± 0.12a	19.03 ± 0.10e	13.71 ± 0.04k	6.29 ± 0.11n	8.86 ± 0.14m	1.65 ± 0.02qr	1.40 ± 0.24r	2.46 ± 0.05p	18.14 ± 0.06 l	0.13 ± 0.01s	0.03 ± 0.02s	
Sinapic acid	22.01 ± 0.07g	23.97 ± 0.03h	24.35 ± 0.07ij	25.91 ± 0.07hi	30.26 ± 0.02g	28.02 ± 0.12hi	19.28 ± 0.10j	23.75 ± 0.02h	17.00 ± 0.13k	11.74 ± 0.14l	12.51 ± 0.03l	13.52 ± 0.13l	12.61 ± 0.05l	
Total phenolics	127 ± 0.17de	144 ± 0.15e	164.47 ± 0.16bc	130.95 ± 0.22d	90.21 ± 0.08j	113.33 ± 0.14 k	123.8 ± 0.14i	109.1 ± 0.10i	73.91 ± 0.21n	81.58 ± 0.15h	103.21 ± 0.15fg	84.86 ± 0.16h	47.74 ± 0.10m	

Values are means ± standard deviations (*n* = 3).

### Tocols, γ-oryzanols, phytosterols, squalene, cholecalciferol and phylloquinone content

RB is an abundant source of tocols (α-, β-, γ-, δ-tocopherol (T), α-, β-, γ-, δ- tocotrienol (T3)), γ-oryzanols, phytosterols and squalene. The changes in tocols content after MWT is shown in Table 3 and chromatographic results are shown in Supplementary Fig. 2 (left side). The vitamin E in the raw RB were γ-T3 (84.86 µg/g), followed by α-T (12.43 µg/g), α-T3 (8.84 µg/g), γ-T (4.29 µg/g), β-T (1.85 µg/g), δ-T3 (1.74 µg/g), and δ-T (0.37 µg/g), respectively. The MWT had positive effects on the tocols content of the RB, especially at 440 W. The changes of the tocols were dependent on the exposure time and microwave power, in which the MWT at 440 W for 2.5 min obtained the highest contents of total tocols (367.09 µg/g, equivalent to 2.6-fold increase from the control of 101.95 µg/g).

**Table 3:** Tocols content (*α*-*, β*-*, γ*- and *δ*-tocopherol (T) and *α*-*, β*-*, γ*- and *δ*-tocotrienol (T3)) of rice bran treated in microwave oven (µg/g).

Compound	Control	130 W							260 W					
		0.5 min	1 min	1.5 min	2 min	2.5 min	3 min	5 min	0.5 min	1 min	1.5 min	2 min	2.5 min	3 min
**Tocol**														
*α*-T	12.43 ± 0.23o	6.50 ± 1.49c	6.76 ± 0.21g	10.38 ± 0.18a	13.13 ± 0.26n	9.81 ± 0.30k	12.42 ± 0.65p	19.66 ± 0.06pq	21.65 ± 0.13d	21.62 ± 0.13b	10.23 ± 0.08s	3.38 ± 0.02h	16.56 ± 0.48g	10.33 ± 0.52n
*β*-T	1.85 ± 0.11rs	1.12 ± 0.13n	1.46 ± 0.19t	2.04 ± 0.13e	3..02 ± 0.07g	2.43 ± 0.16m	1.70 ± 0.12p	1.65 ± 0.08l	1..85 ± 0.14p	1.94 ± 0.09o	2.32 ± 0.11k	3.24 ± 0.09v	3.48 ± 0.22w	2.02 ± 0.37u
*γ*-T	4.29 ± 0.21l	2.66 ± 0.33q	1.55 ± 0.50q	3.13 ± 0.22m	4.98 ± 0.77k	4.80 ± 0.58n	5.48 ± 0.27l	2.95 ± 0.03g	0.74 ± 0.14f	2.53 ± 0.09f	3.52 ± 0.53m	2.78 ± 0.83b	2.53 ± 0.02h	3.44 ± 0.06m
*δ*-T	0.37 ± 0.00k	0.69 ± 0.01pq	0.70 ± 0.04r	0.89 ± 0.03no	0.72 ± 0.04ij	0.69 ± 0.05j	0.39 ± 0.01g	0.29 ± 0.01op	0.73 ± 0.06s	0.82 ± 0.03q	0.98 ± 0.05m	1.04 ± 0.20t	1.45 ± 0.09q	1.37 ± 0.11mn
*α*-T3	8.84 ± 0.07e	9.65 ± 0.13kl	9.78 ± 0.03mn	8.07 ± 0.08hi	7.43 ± 0.12jk	7.40 ± 0.09kl	6.91 ± 0.40lm	5.93 ± 0.28o	10.48 ± 0.39h	10.62 ± 0.12ij	10.77 ± 0.07kl	10.70 ± 0.07l	8.96 ± 0.09no	7.36 ± 0.05p
*β*-T3	ND	ND	ND	ND	ND	ND	ND	ND	ND	ND	ND	ND	ND	ND
*γ*-T3	84.86 ± 0.20h	92.34 ± 0.61c	99.85 ± 0.48g	101.10 ± 0.72k	112.59 ± 0.23m	109.05 ± 0.16op	97.23 ± 0.66t	50.17 ± 0.35f	57.95 ± 0.51o	89.79 ± 0.62pq	161.20 ± 0.37v	164.69 ± 0.22v	166.26 ± 1.57w	59.97 ± 0.01s
*δ*-T3	1.74 ± 0.93m	2.83 ± 0.08l	2.04 ± 0.25t	3.98 ± 0.02j	4.97 ± 0.08gh	2.91 ± 0.93hi	2.56 ± 0.86jk	2.34 ± 0.39t	2.51 ± 0.63o	2.66 ± 0.21n	4.59 ± 1.12rs	5.09 ± 0.14g	4.74 ± 0.22pq	1.53 ± 0.31s
Total tocols	101.95 ± 0.87n	109.29 ± 0.46k	115.38 ± 0.88t	119.21 ± 0.71i	130.69 ± 0.52h	127.28 ± 0.91i	114 ± 0.53l	63.33 ± 0.75st	72.41 ± 0.07o	108.36 ± 0.79lm	183.38 ± 0.97rs	187.5 ± 0.72q	187.42 ± 0.64p	75.69 ± 0.73r

Compound	440 W					620 W				880 W				
	0.5 min	1 min	1.5 min	2 min	2.5 min	0.5 min	1 min	1.5 min	2 min	0.5 min	1 min	1.5 min	2 min	
**Tocol**														
*α*-T	59.25 ± 0.33f	16.18 ± 0.02s	9.51 ± 0.36r	31.76 ± 0.46q	79.90 ± 0.26e	8.19 ± 0.08l	10.30 ± 0.26r	8.23 ± 0.02j	8.16 ± 0.05k	16.61 ± 0.34m	26.63 ± 0.12k	33.37 ± 0.25i	14.73 ± 0.18k	
*β*-T	2.62 ± 0.14b	3.27 ± 0.12h	3.45 ± 0.14c	3.62 ± 0.15d	1.70 ± 0.12a	2.24 ± 0.07r	2.41 ± 0.08q	2.71 ± 0.04j	5.23 ± 0.01st	2.14 ± 0.03o	2.44 ± 0.49 l	1.39 ± 0.03f	1.19 ± 0.13i	
*γ*-T	14.36 ± 0.28b	3.71 ± 0.36i	5.35 ± 0.63o	11.36 ± 0.37d	14.18 ± 0.61a	5.66 ± 0.03p	3.87 ± 0.07m	6.21 ± 0.37p	6.07 ± 0.06p	6.72 ± 0.01h	5.12 ± 0.09e	6.29 ± 0.07c	5.93 ± 0.07j	
*δ*-T	0.76 ± 0.02a	1.35 ± 0.03lm	1.46 ± 0.02gh	1.07 ± 0.14b	4.00 ± 0.06a	0.52 ± 0.07fg	1.25 ± 0.08l	1.02 ± 0.19de	0.89 ± 0.04de	0.65 ± 0.03c	1.42 ± 0.03hi	0.89 ± 0.03d	0.61 ± 0.07ef	
*α*-T3	18.04 ± 0.06a	8.27 ± 0.12o	9.74 ± 0.05o	11.92 ± 0.12c	12.04 ± 0.29b	8.49 ± 0.18jk	5.49 ± 0.17g	8.51 ± 0.15f	7.64 ± 0.73m	8.60 ± 0.06d	5.68 ± 0.12h	7.97 ± 0.10f	7.28 ± 0.14jk	
*β*-T3	ND	ND	ND	ND	ND	ND	ND	ND	ND	ND	ND	ND	ND	
*γ*-T3	91.19 ± 0.30a	99.98 ± 0.87r	188.07 ± 0.39n	209.99 ± 0.80b	238.61 ± 0.39e	119.28 ± 0.06s	196.60 ± 0.27x	126.72 ± 0.03l	267.13 ± 0.02j	114.76 ± 0.50i	131.55 ± 0.13d	122.87 ± 0.38l	117.09 ± 0.84j	
*δ*-T3	4.23 ± 0.50a	4.48 ± 0.63p	6.73 ± 0.01m	6.57 ± 0.32b	16.66 ± 0.64a	2.18 ± 0.08fg	2.42 ± 0.26k	5.70 ± 0.32i	4.28 ± 0.04l	2.67 ± 0.36de	3.34 ± 0.06c	3.82 ± 0.04d	4.20 ± 0.35ef	
Total tocols	190.45 ± 0.97b	137.24 ± 0.02p	224.31 ± 0.26no	276.29 ± 0.03c	367.09 ± 0.05a	146.56 ± 0.06h	222.34 ± 0.36m	159.10 ± 0.99i	299.40 ± 0.04j	152.15 ± 0.43f	176.18 ± 0.36d	176.6 ± 0.21e	151.03 ± 0.08g	

*ND *non detectable.

Values are means ± standard deviations (*n* = 3).

The results for other functional compounds are shown in Table 4, and the chromatographic result is shown in Supplementary Fig. 2 (right side). These include γ-oryzanols, a fundamental substance with several health-beneficial effects, such as anti-oxidant activity, anticarcinogenic, and antidiabetic^17,18^. The main γ-oryzanols in the raw RB was 24-methylene cycloartanyl ferulate (24-MCFer) (716.55 μg/g), followed by cycloartenyl ferulate (CycloFer) (442.77 μg/g), campesteryl ferulate (CampFer) (270.05 μg/g), and β-sitosteryl ferulate (β-SitFer) (119.94 μg/g) with the total γ-oryzanol content of control at 1549.31 μg/g. The current study showed an enhancement of γ-oryzanols after MWT. The optimum exposure power was 260 W, which the CycloFer, 24-MCFer, CampFer, and β-SitFer increased 1.3, 2.4, 0.6, and 1.4 folds than those of the control, respectively. The MWT of KDML 105 RB in this study showed a 1.6-fold increase of total γ-oryzanols while the parboiled and steamed of Sona masuri RB showed 0.7 and 0.4-fold increase^13^. Generally, the MWT contributed to the positive changes in total phytosterol contents.

**Table 4:** Content of γ-oryzanols, phytosterols, squalene, cholecalciferol and phylloquinone of rice bran treated in microwave oven (µg/g).

Compound	Control	130 W							260 W					
		0.5 min	1 min	1.5 min	2 min	2.5 min	3 min	5 min	0.5 min	1 min	1.5 min	2 min	2.5 min	3 min
γ-Oryzanols														
CycloFer	442.77x	503.13t	767.16s	891.24 m	917.79k	986.20g	978.14h	354.21w	560.75r	872.13e	944.85y	1009.27l	1028.40b	988.29f
24-MCFer	716.55y	727.02w	1140.1o	1295.97t	1329.05s	1567.18j	1576.90i	564.97z	1295.6r	1301.5g	1603.5v	2403.89a	1708.46f	1553.90b
CampFer	270.05n	287.72o	301.08k	349.46g	362.85f	394.21b	405.04a	92.83p	320.27i	401.59a	411.00q	437.66h	404.93a	394.31b
β-SitFer	119.94v	124.34s	145.19r	166.62o	168.58n	186.79j	193.50h	199.69y	147.66q	156.59t	212.85x	222.59w	284.88k	183.93l
Total γ-oryzanols	1549.31y	1642.21w	912.35r	2703.39q	2778.27o	3134.38i	3153.58g	1211.70z	2324.07t	2731.81j	3172.2x	4073.35a	3426.67e	3120.43k
Phytosterols														
STIG + CAMP	827.25c	845.37g	994.64i	1015.43q	1032.00f	933.38r	789.98l	784.37s	922.34n	1012.23e	1040.53k	1254.46q	943.51g	845.36a
β-SIT	424.76kl	645.21hi	1075.23m	1101.95m	1168.04m	1199.7m	1225.8m	1904.84g	1124.05f	1224.40ghi	1136.76e	1255.04ef	913.65g	1590.62cd
Total Phytosterols	1252.01o	1490.58n	2069.87w	2117.38y	2200.04s	2133.08x	2015.78u	2689.21m	2046.39j	2236.63l	2177.29h	2509.5i	1857.16 k	2435.98c
Squalene	99.55g	109.86h	225.23i	249.32d	147.15f.	143.41s	112.71k	80.22h	199.30i	246.29e	253.14r	225.29y	152.72w	42.89x
Cholecalciferol	3.01h	3.37i	4.51i	7.29e	4.48gh	3.46m	2.63n	1.25q	3.48q	4.80jk	8.92b	5.59k	4.54i	3.86l
Phylloquinone	2.45f	2.55p	2.83q	3.43q	8.28g	4.54m	4.09n	0.00a	2.74d	3.60o	4.29j	10.94c	4.16s	0.56t

Compound	440 W					620 W				880 W				
	0.5 min	1 min	1.5 min	2 min	2.5 min	0.5 min	1 min	1.5 min	2 min	0.5 min	1 min	1.5 min	2 min	
γ-Oryzanols														
CycloFer	775.00a	996.83p	1000.7i	939.12j	485.39u	999.41d	985.80g	891.10o	880.79o	788.00v	846.34q	888.99c	724.41n	
24-MCFer	1330.1e	1336.3q	1976.30p	1643.10h	1380.50c	1520.57b	1555.78b	1562.66l	1496.49m	720.72u	725.77x	790.17d	692.80n	
CampFer	321.71l	376.29 g	419.77d	368.00e	312.11j	306.56c	362.63 cd	361.530f.	360.22f	245.20m	348.98g	379.80d	340.24f	
β-SitFer	165.13p	187.34i	241.66f	198.43v	136.34u	217.69e	251.99b	207.71d	190.13d	204.08g	277.63a	113.57c	109.49m	
Total γ-oryzanols	2591.94c	2896.76p	2637.73n	3148.65f	2314.41s	3044.23b	3156.20h	3023.00l	2927.63l	1237.28v	2198.72u	2172.53d	1866.94m	
Phytosterols														
STIG + CAMP	999.08m	1369.87o	1136.94p	949.79j	774.93t	941.37h	1237.28b	926.85d	918.37k	871.77u	940.31d	791.65t	706.28r	
β-SIT	1130.20ij	1689.69m	1524.97jk	1503.14jk	856.46m	1412.31de	1771.08bc	1158.68gh	1163.10f	1244.92lm	1309.17a	1149.35b	1009.20bc	
Total Phytosterols	2129.28p	3059.56v	2661.91r	2452.93q	1631.39z	2353.68f	3008.36b	2085.53g	2081.47g	2116.69t	2249.48a	1941.00d	1715.48e	
Squalene	162.70q	271.11m	303.87j	278.94o	135.66u	142.25t	270.39b	264.24c	264.04c	129.57v	109.33l	83.89a	74.97p	
Cholecalciferol	3.98m	4.86jk	12.89l	4.81op	1.54p	6.64fg	9.23d	7.07j	1.97n	14.15a	9.84c	3.78f	1.03o	
Phylloquinone	3.53r	4.48op	5.71l	10.39e	2.17h	5.06k	6.52i	4.39n	4.14n	2.40k	11.91a	2.40b	2.18h	

Values are means ± standard deviations (*n* = 3).

The highest total phytosterol content was found in the RB treated at 440 W for 1 min (3059.56 µg/g), which increased 1.4 folds from the control (1252.01 µg/g). In most cases, microwave-treated RB showed higher levels of β-sitosterol (β-SIT) than the raw RB (424.76 μg/g). The highest content of β-SIT was found in the RB treated at 130 W for 5 min (1904.84 μg/g) and increased 3.5 folds from the control. The highest stigmasterol and campesterol (STIG + CAMP) content was found in the RB treated at 440 W for 1 min (1369.87 μg/g) which increased 0.7 folds as compared to the control (827.25 μg/g). The increase in MW power 130 W to 440 W at exposure time 0.5, 1, and 1.5 min contributed to a gradual increase in the STIG + CAMP content. However, a decrease in STIG + CAMP amount as compared to the control was observed at 880 W for 1.5 and 2 min as well as 130 W for 3 to 5 min. This indicated that the high power and long exposure time in the MWT caused significant damage to the phytochemicals content.

The content of squalene (99.55 µg/g), cholecalciferol (3.01 µg/g), and phylloquinone (2.45 µg/g) in the control showed improvements after the MWT. Roasting at 440 W for 1.5 min obtained the highest content of squalene (303.89 µg/g, increased by 2.0 folds, and roasting at 880 W provided the highest content of cholecalciferol (14.15 µg/g, increased by 3.7 folds) and phylloquinone (11.91 µg/g, increased by 3.9 folds). The impact of exposure time on phylloquinone determination exhibited the same trend as the effect of time on squalene content. Pokkanta et al., (2019) reported that RBs were an abundant source of phytosterols (stigmasterol, campesterol and β-sitosterol) and squalene^19^.

Based on our results, the changes of phytochemicals when exposed to MWT with increasing power and exposure time share the same trend. The phytochemical content in RBs after MWT proportionally increased with increasing MW power and exposure time until it reaches its highest value. Sequentially, a decrease in phytochemical content was observed for MWT at high power and long exposure time. This could be because the phytochemicals in plant cell walls, such as phenolic compounds, dissolve due to the breakage of the bonds that connect them. Solubility of the phytochemical increased as a result of its dissolution in cell tissue, increasing the released phytochemical^20^. The heat generated from the MWO can inactivate enzymes such as lipase and oxidase, causing deterioration of the phytochemicals. The antioxidant activity was increased partly due to the formation of the Maillard reaction products, an antioxidant in foods^21^. On the other hand, after each phytochemical increased to its highest content, it began to degrade. The MWT at high power and long operation times can lead to the elevated temperature of the sample. The generated heat acts particularly on polar bonds of the compounds, contributing to chemical reactions such as oxidation, dehydration, structural changes, and esterification that can react or transform secondary plant metabolites into other structures^22^. Furthermore, excessive microwave exposure can degrade phytochemicals of natural products due to the electromagnetic force of microwave, thermal-accelerated oxidative deterioration, especially in heat-sensitive substances (e.g., polyphenols)^23^.

In general, MWT could improve the nutrients of food samples, however, the appropriate MW power and exposure time are required for different crop material to retain high amounts of phytochemicals. The results found different optimum conditions for the content of γ-oryzanols (260 W for 2 min), tocols (440 W for 2.5 min), phytosterols (440 W for 1 min), squalene (440 W for 1.5 min), cholecalciferol, and phylloquinone (880 W for 1 min). However, the MWT at 440 W for 2.5 min was concluded as the best overall condition, which provided the highest content of the studied bioactive compounds and antioxidant activity.

## Conclusions

The study revealed that the MWT increased antioxidant activities and amounts of released bioactive compounds from the RB. The MWT was able to increase the capability of the phytochemical compounds to be released from their bound structures. The MWT required very little time, therefore, enabling the preservation of nutraceutical values and properties of the RB. The long exposure time and high power in the microwave process might cause degradation of the nutrients. The findings suggested that the MWT could be a powerful tool for the stabilization, enhancement of usability, and retainment of RB phytochemicals.

## Material and methods

### Plant materials

KDML 105 (the most popular aromatic rice variety in Thailand) RB sample was requested and permitted from the Suphanburi Rice Research Center (a government office of the Rice Department, Ministry of Agriculture and Cooperative), Thailand in December 2019. The RB was sieved through 60-mesh, packed in a ziplock bag, and stored at -10 °C until the day of sample preparation.

### Chemicals

Standards of phenolics, γ-oryzanols, phytosterols, squalene, cholecalciferol, phylloquinone were purchased from Tokyo Chemical Industry Co., Ltd. (Tokyo, Japan). Standard tocols, Folin-Ciocalteu reagent, and 1,1-diphenyl-2-picrylhydrazyl (DPPH) were purchased from Sigma-Aldrich Co., Ltd, (Darmstadt, Germany) and Eisai Food & Chemical Co., Ltd. (Tokyo, Japan). The other chemicals used were of analytical grade from RCI Labscan Co., Ltd. (Bangkok, Thailand).

## Methods

The study complies with local and national guidelines.

### Microwave stabilization

A MWO (R-2200F-S, cavity of 30.6 × 30.7 × 20.8 cm, Sharp, Thailand) capable of generating power of 880 W at 2450 MHz was used for the roasting experiments. A petri dish with a 100 mm diameter containing the RB sample (10 g) was placed in the middle of the rotary plate of the MW oven (i.d. 28 cm). The RB samples were heated with 130, 260, 440, 620, and 880 W and exposure duration of 0.5, 1.0, 1.5, 2.0, 2.5, 3.0, and 5.0 min.

### Spectrometry analysis of phenolics, flavonoids, and antioxidant activity in RB

RBs (0.5 g) were extracted with 5.00 mL of 80% methanol under sonication for 1 h. The extraction solvent was chosen because it is proven to be the most effective extraction solvent for phenolics and antioxidant activity in rice^24^. Sonication was used to maximize extraction efficacy of the targeted compounds^25^. The resulting solution was centrifuged at 3500 rpm for 10 min, and the supernatant was filtered through a 0.45 μm nylon filter. The resulting extract was subjected to determination of phenolics^26^, flavonoids^27^, and antioxidant activity^28^ with a UNICO 2150-UV Spectrophotometer.

### HPLC analysis of individual phenolic, tocols, γ-oryzanols, phytosterols, squalene, cholecalciferol and phylloquinone

Two HPLC systems were employed. The first system was applied for the analysis of the eleven phenolic compounds^29^. Phenolic compounds were extracted with the same method used in the spectrometric analysis. The system utilized a Kinetex C18 column (150 × 4.6 mm; 2.6 µm, Phenomenex) and a gradient elution system consisting of water/acetic acid (99:1, v/v) as mobile phase A and water/acetonitrile/acetic acid (67:32:1, v/v/v) as mobile phase B. The phenolics were detected at 275 nm. The other HPLC system was for analysis of the other functional phytochemicals (total of seventeen compounds)^19^. RBs (0.30 g) were extracted with methanol (3.00 mL) for 5 min, and the extracted RBs were then re-extracted with dichloromethane (3.00 mL) and hexane (3.00 mL), respectively. Supernatants of these three solvents were combined, evaporated, and re-dissolved with dichloromethane before analysis. The HPLC system employed a Kinetex PFP column (4.6 × 250 mm, 5 µm, Phenomenex) and a mobile phase of methanol and water. A fluorescent detector was set at 294 nm (excitation) and 326 nm (emission) for detection of tocols, and a variable wavelength detector was set to detect cholecalciferol at 265 nm (0–8 min), phytosterols and squalene at 210 nm (8–18 min), and phylloquinone and γ-oryzanols at 328 nm (18–30 min).

### Statistical analysis

Quantitative data were expressed as the mean ± standard deviation (n = 3). Statistical analysis in this study was analyzed using one-way ANOVA with RStudio version 1.2.5042. Differences are statistically significant at *P* < 0.05.

## Supplementary Information

Supplementary Information.
