# Supplementary material for: Microwave treatment of rice bran and its effect on phytochemical content and antioxidant activity
Source: Sci Rep. 2022 May 11;12:7708. doi: 10.1038/s41598-022-11744-1 (PMC9095620; doi:10.1038/s41598-022-11744-1)
Supplement: Supplementary file 1 — Supplementary Information. [file 41598_2022_11744_MOESM1_ESM.docx]

**Microwave Treatment of Rice Bran and Its Effect on Phytochemical Content and Antioxidant Activity**

Piramon Pokkanta^1,2^, Jitkunya Yuenyong^1^, Sugunya Mahatheeranont^1,3,4^,

Sudarat Jiamyangyuen^5^ & Phumon Sookwong^1,3,4*^

^1^Rice and Cereal Chemistry Research Laboratory, Department of Chemistry, Faculty of Science, Chiang Mai University, Chiang Mai 50200, Thailand

^2^PhD’s Degree Program in Chemistry, Faculty of Science, Chiang Mai University, Chiang Mai 50200, Thailand

^3^Research Center on Chemistry for Development of Health Promoting Products from Northern Resources, Chiang Mai University, Chiang Mai 50200, Thailand

^4^Center of Excellence for Innovation in Chemistry, Faculty of Science, Chiang Mai University, Chiang Mai 50200, Thailand

^5^Rice and Bioactive Compound Analysis, Department of Agro-Industry, Faculty of Agriculture, Natural Resources and Environment, Naresuan University, Phitsanulok 65000, Thailand.

*Corresponding author E-mail: phumon.s@cmu.ac.th


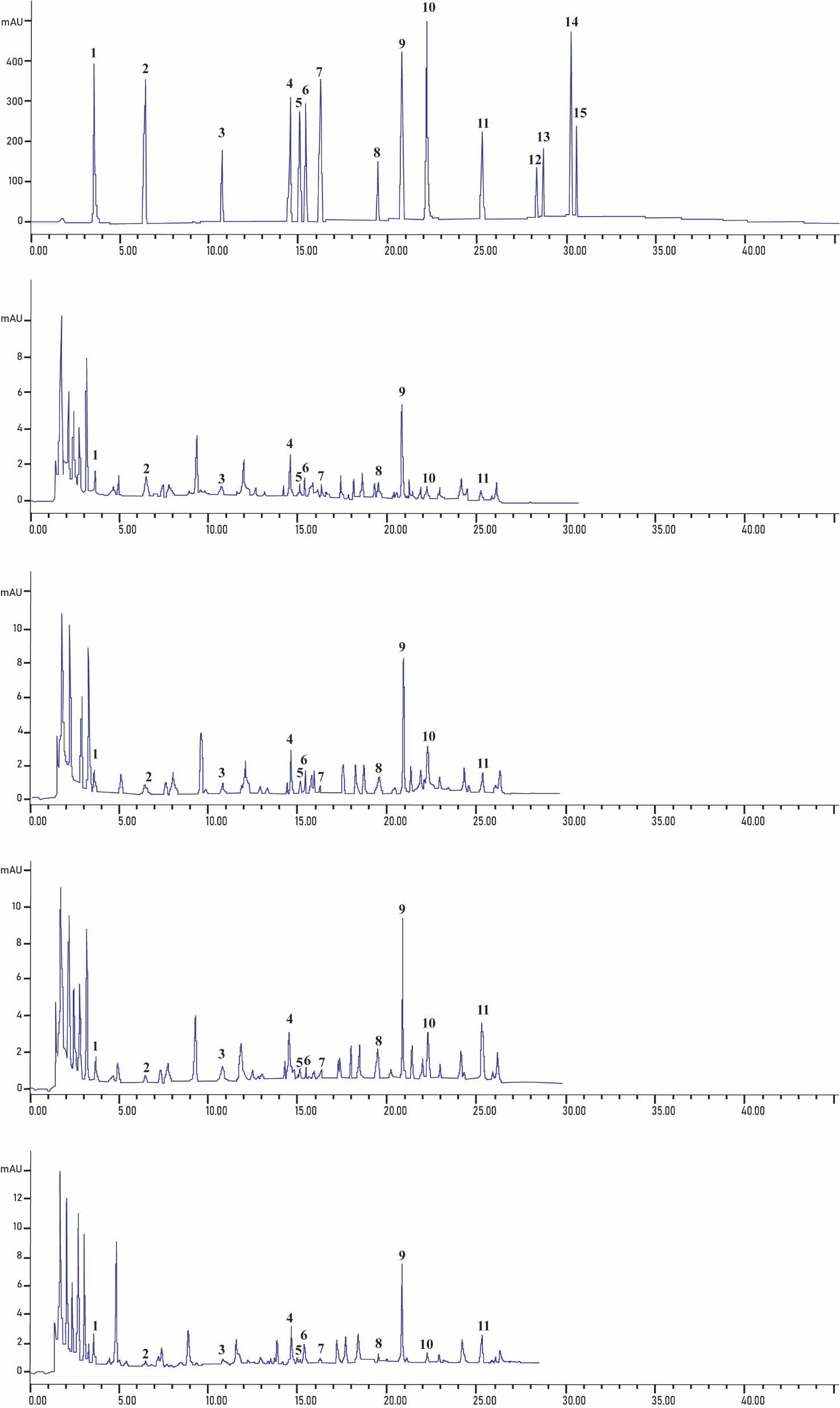


**Figure 1**

**A )**

**B )**

**C )**

**D )**

**E )**

min

min

min

min

min


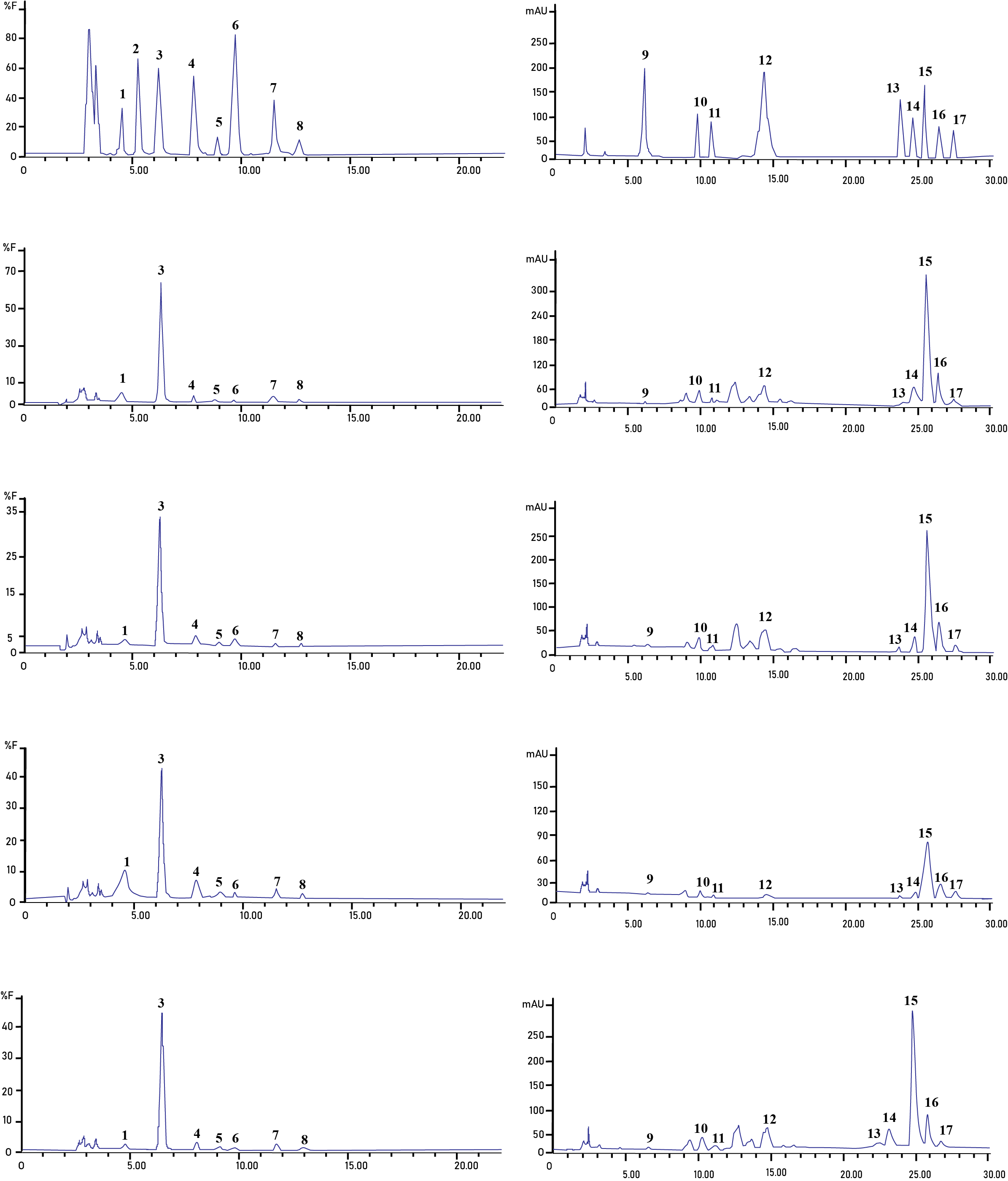


**Figure 2**

Variable wavelength

Variable wavelength

Variable wavelength

Variable wavelength

Variable wavelength

Fluorescence

Fluorescence

Fluorescence

Fluorescence

Fluorescence

**A )**

**B )**

**C )**

**D )**

**E )**

min

min

min

min

min

min

min

min

min

min


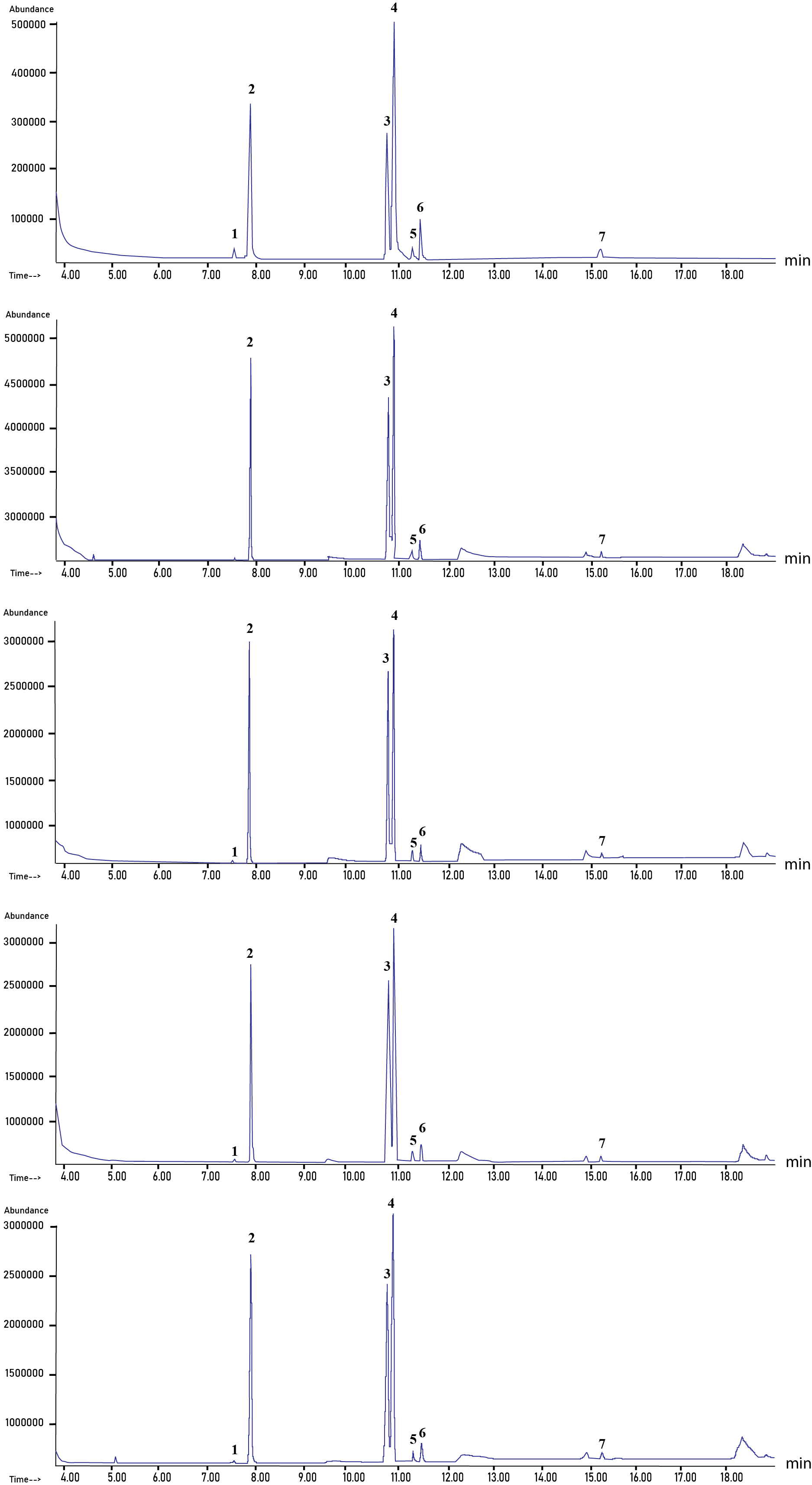


**Figure 3**

**A )**

**B )**

**C )**

**D )**

**E )**

**Figure Captions**

Figure 1. HPLC chromatograms of eleven phenolic compounds. A) Standard, B) Raw KDML 105 rice bran, C) Microwave treatment at 260 W for 1.5 min, D) Microwave treatment at 440 W for 1.5 min, and E) Microwave treatment at 800 W for 1.5 min. Peak identification: 1 = gallic acid, 2 = protocatechuic acid, 3 = 4-hydroxybenzoic acid, 4 = catechin, 5 = vanillic acid, 6 = chlorogenic acid, 7 = caffeic acid, 8 = kaempferol, 9 = epigallocatechin, 10 = trans-*p*-coumaric acid, and 11 = sinapic acid

Figure 2. HPLC chromatograms of bioactive compounds. A) Standard, B) Raw KDML 105 rice bran, C) Microwave treatment at 260 W for 2.0 min, D) Microwave treatment at 440 W for 2.5 min, and E) Microwave treatment at 800 W for 1.5 min. Peak identification: Peak identification: 1 = δ-T3, 2 = β-T3, 3 = γ-T3, 4 = α-T3, 5 = δ-T, 6 = β-T, 7 = γ-T, 8 = α-T, 9 = cholecalciferol, 10 = stigmasterol + campesterol, 11 = β-sitosterol, 12 = squalene, 13 = phylloquinone, 14 = CycloFer, 15 = 24-MCFer, 16 = CampFer, and 17 = β-SitFer

Figure 3. GC chromatograms of fatty acid methyl esters (FAME). A) Standard, B) Raw KDML 105 rice bran, C) Microwave treatment at 260 W for 3.0 min, D) Microwave treatment at 440 W for 0.5 min, and E) Microwave treatment at 880 W for 1.5 min. Peak identification: 1 = Methyl palmitoleate (FAME of palmitoleic acid), 2 = methyl palmitate (FAME of palmitic acid), 3 = methyl linoleate (FAME of linoleic acid), 4 = methyl oleate (FAME of oleic acid), 5 = methyl linolenate (FAME of linolenic acid), 6 = methyl stearate (FAME of stearic acid), and 7 = methyl arachidate (FAME of arachidic acid)
